# Supplementary material for: Resonance Raman Spectro-Electrochemistry to Illuminate Photo-Induced Molecular Reaction Pathways
Source: Molecules. 2019 Jan 10;24(2):245. doi: 10.3390/molecules24020245 (PMC6358810; doi:10.3390/molecules24020245)
Supplement: Supplementary file 1 [file molecules-24-00245-s001.pdf]

Supporting information

Resonance Raman Spectro-Electrochemistry to Illuminate Photo-Induced Molecular Reaction Pathways

Linda Zedler[1], Sven Krieck[2], Stephan Kupfer[3] and Benjamin Dietzek[1,3,*]

[1] Leibniz Institute of Photonic Technology Jena, Albert-Einstein-Strasse 9, 07745 Jena, Germany; linda.zedler@leibniz-ipht.de
[2] Institute of Inorganic and Analytical Chemistry, Friedrich-Schiller-University Jena, Humboldtstrasse 8, 07743 Jena, Germany; sven.krieck@uni-jena.de
[3] Institute of Physical Chemistry and Abbe Center of Photonics, Friedrich-Schiller-University Jena, Helmholtzweg 4, 07743 Jena, Germany; stephan.kupfer@uni-jena.de
***** Correspondence: benjamin.dietzek@uni-jena.de; Tel.: +49-3641-948360

Computational evaluation of the UV-vis-SEC

The main alternations in the absorption spectra upon single reduction (Figure 2B) are associated with IL and MLCT transitions. The electrochemically generated electron residing within the LUMO of the non-reduced complex is photo-excited in case of the energetically low-lying IL transitions (D_7, D_{13}, D_{14}, and partially D_{22}) between 756 (1.64 eV) and 488 nm (2.54 nm). The doublet transitions at higher energies, i.e., between 463 (2.68 eV) and 345 nm (3.60 eV), are mainly of MLCT nature (D_{25}, D_{35} and D_{57}), while brighter IL $\pi_{tpy} \rightarrow \pi^*_{tpy}$ transitions, e.g., state D_{63} and D_{80}, are found at even higher energies (Table S1). In summary and according to the calculations, the long wavelength absorption feature (> 700 nm) as well as the new absorption band centered at around 630 nm of the single-reduced form are dominated by $\pi^*_{tpy} \rightarrow \pi^*_{tpy}$ IL transitions, i.e., D_7 at 756 nm and D_{13} and D_{14} at 568 and 557 nm, respectively. Additionally, the shoulder observed at around 536 nm in the experimental spectrum (Figure 1B) can be assigned to a superposition of excitations into the states D_{22} and D_{25}. The state D_{22} (488 nm) presents a pronounced mixture of IL ($\pi^*_{tpy} \rightarrow \pi^*_{tpy}$) and ^{2}MLCT transitions. D_{25} (463 nm) is of pure MLCT nature, while the charge density is exclusively shifted upon photoexcitation towards the non-reduced terpyridine ligand in $[(tbtpy)Ru(tbtpy^{\bullet})]^+$. Finally, the spectral changes at higher excitation energy around 414 nm can be mainly associated to the population of the D_{35} (405 nm) and D_{57} (345 nm) states of mixed MLCT/IL character.

In the following, the observed spectral changes are discussed with respect to both possible scenarios. Considering the formation of the energetically slightly favored doubly reduced triplet species the experimentally observed changes at around 800 nm can be related to the excitation into the doubly degenerated ^{3}IL ($\pi^*_{terpy} \rightarrow \pi^*_{terpy}$) states, T_9, and T_{10}, at 758 and 779 nm (Figure 2D). The IL and MLCT states T_{15} and T_{16} (584 and 581 nm) may be assigned to the broad absorption band of the experimental spectrum at around 516 nm. Finally, the spectral variations upon double reduction centered at 444 nm can be attributed to the most intense absorption band at 478 nm in the theoretical spectrum, which stems from a superposition of three MLCT states, namely T_{32}, T_{33}, and T_{35}.

Alternatively, the doubly reduced singlet species is considered, where the low-lying $\pi^*_{tpy} \rightarrow \pi^*_{tpy}$ IL state, S_7 at 726 nm, is found at a slightly higher excitation energy than the analogous ^{3}IL states T_9 and T_{10} of the triplet species. Excitation into this S_7 may be associated to the spectral changes in the NIR region, while the variations observed between 470 and 650 nm can be attributed to a superposition of several medium bright singlet excited states (S_{11}, S_{13}, S_{14} S_{16} S_{18}, and S_{20}), which are mainly of MLCT character (see Table S1 of SI). The rising absorption feature at 426 nm can be correlated to the superposition of a bright IL/LMCT (S_{25}) and a bright IL state (S_{26}) at 396 and 390 nm, respectively.

Table S1: Calculated bright electronic excited states for the non-reduced (singlet), singly (doublet), and doubly reduced (singlet and triplet) species of $[(tbterpy)_2Ru]^{2+}$ with the main transitions, excitation energies E^e and wavelengths λ, oscillator strengths f, and the eigen values of $\langle s^2 \rangle$.

**Non-reduced singlet (S=0)**						
State	Transition	Weight / %	E^e / eV	λ / nm	f	$\langle s^2 \rangle$
S_1	$d_{yz}(153) \rightarrow \pi^*_{tpy}(154)$ (MLCT)<br>$d_{yz}(153) \rightarrow \pi^*_{tpy}(155)$ (MLCT)	79<br>19	2.55	486	0.019	0.000
S_2	$d_{yz}(153) \rightarrow \pi^*_{tpy}(155)$ (MLCT)<br>$d_{yz}(153) \rightarrow \pi^*_{tpy}(154)$ (MLCT)	79<br>19	2.55	486	0.019	0.000
S_5	$d_{yz}(153) \rightarrow \pi^*_{tpy}(156)$ (MLCT)<br>$d_{xz}(152) \rightarrow \pi^*_{tpy}(155)$ (MLCT)<br>$d_{xy}(151) \rightarrow \pi^*_{tpy}(154)$ (MLCT)<br>$d_{xz}(152) \rightarrow \pi^*_{tpy}(154)$ (MLCT)<br>$d_{xy}(151) \rightarrow \pi^*_{tpy}(155)$ (MLCT)	31<br>21<br>19<br>14<br>14	2.76	449	0.050	0.000
S_7	$d_{yz}(153) \rightarrow \pi^*_{tpy}(156)$ (MLCT)<br>$d_{xz}(152) \rightarrow \pi^*_{tpy}(156)$ (MLCT)<br>$d_{xy}(151) \rightarrow \pi^*_{tpy}(154)$ (MLCT)	54<br>9<br>8	2.89	429	0.171	0.000
S_8	$d_{xz}(152) \rightarrow \pi^*_{tpy}(156)$ (MLCT)<br>$d_{xy}(151) \rightarrow \pi^*_{tpy}(156)$ (MLCT)	69<br>27	2.90	428	0.029	0.000
S_9	$d_{xy}(151) \rightarrow \pi^*_{tpy}(156)$ (MLCT)<br>$d_{xz}(152) \rightarrow \pi^*_{tpy}(156)$ (MLCT)<br>$d_{yz}(153) \rightarrow \pi^*_{tpy}(156)$ (MLCT)	65<br>19<br>11	2.90	428	0.049	0.000
S_{10}	$d_{xz}(152) \rightarrow \pi^*_{tpy}(157)$ (MLCT)	96	2.92	424	0.025	0.000
S_{11}	$d_{xy}(151) \rightarrow \pi^*_{tpy}(157)$ (MLCT)	94	2.92	424	0.035	0.000
S_{27}	$d_{yz}(150) \rightarrow \pi^*_{tpy}(154)$ (IL)<br>$d_{yz}(153) \rightarrow \pi^*_{tpy}(160)$ (MLCT)	75<br>11	4.09	303	0.275	0.000
S_{28}	$\pi_{tpy}(150) \rightarrow \pi^*_{tpy}(155)$ (IL)<br>$d_{yz}(153) \rightarrow \pi^*_{tpy}(159)$ (MLCT)	76<br>11	4.09	303	0.280	0.000
**Singly reduced doublet (S=1/2)**						
State	Transition	Weight / %	E^e / eV	λ / nm	f	$\langle s^2 \rangle$
D_7	$\pi^*_{tpy}(154\alpha) \rightarrow \pi^*_{tpy}(161\alpha)$ (IL)<br>$\pi^*_{tpy}(154\alpha) \rightarrow \pi^*_{tpy}(160\alpha)$ (IL)	74<br>21	1.64	756	0.021	0.782
D_{13}	$\pi^*_{tpy}(154\alpha) \rightarrow \pi^*_{tpy}(164\alpha)$ (IL)	92	2.18	568	0.059	0.788
D_{14}	$\pi^*_{tpy}(154\alpha) \rightarrow \pi^*_{tpy}(155\alpha)$ (IL)<br>$\pi^*_{tpy}(153\beta) \rightarrow \pi^*_{tpy}(154\beta)$ (MLCT)	58<br>29	2.22	557	0.016	0.813
D_{22}	$\pi^*_{tpy}(154\alpha) \rightarrow \pi^*_{tpy}(156\alpha)$ (IL)<br>$d_{xz}(151\beta) \rightarrow \pi^*_{tpy}(155\beta)$ (MLCT)	53<br>43	2.54	488	0.062	0.768
D_{25}	$d_{yz}(153\alpha) \rightarrow \pi^*_{tpy}(157\alpha)$ (MLCT)<br>$d_{yz}(153\beta) \rightarrow \pi^*_{tpy}(156\beta)$ (MLCT)<br>$d_{xy}(152\beta) \rightarrow \pi^*_{tpy}(156\beta)$ (MLCT)<br>$d_{yz}(153\beta) \rightarrow \pi^*_{tpy}(157\beta)$ (MLCT)<br>$d_{xz}(152\alpha) \rightarrow \pi^*_{tpy}(155\alpha)$ (MLCT)<br>$d_{xy}(151\alpha) \rightarrow \pi^*_{tpy}(156\alpha)$ (MLCT)	23<br>17<br>14<br>11<br>7<br>7	2.68	463	0.061	0.876
D_{35}	$d_{xy}(152\beta) \rightarrow \pi^*_{tpy}(157\beta)$ (MLCT)<br>$\pi^*_{tpy}(154\alpha) \rightarrow \pi^*_{tpy}(171\alpha)$ (IL)<br>$d_{yz}(153\alpha) \rightarrow \pi^*_{tpy}(157\alpha)$ (MLCT)	40<br>30<br>8	3.06	405	0.059	0.906
D_{57}	$d_{xz}(151\beta) \rightarrow \pi^*_{tpy}(159\beta)$ (MLCT)<br>$d_{xz}(152\alpha) \rightarrow \pi^*_{tpy}(158\alpha)$ (MLCT)	39<br>38	3.60	345	0.036	0.795

	d_{xz}(152α) → π^*_{tpy}(159α) (MLCT)	13				
D_{63}	π_{tpy}(150β) → π^*_{tpy}(157β) (IL)<br>π_{tpy}(150β) → π^*_{tpy}(156β) (IL)<br>d_{yz}(153β) → π^*_{tpy}(160β) (MLCT)	33<br>25<br>10	3.71	334	0.141	0.948
D_{80}	π_{tpy}(149β) → π^*_{tpy}(154β) (IL)<br>π_{tpy}(149α) → π^*_{tpy}(155α) (IL)	46<br>44	4.13	300	0.452	0.775
**Doubly reduced singlet (S=0)**						
State	Transition	Weight / %	E^e / eV	λ / nm	f	$\langle s^2 \rangle$
S_7	π^*_{tpy}(154) → π^*_{tpy}(161) (IL)	92	1.71	726	0.023	0.000
S_{11}	π^*_{tpy}(154) → π^*_{tpy}(164) (IL)	91	2.24	554	0.036	0.000
S_{13}	d_{xy}(152) → π^*_{tpy}(156) (MLCT)	85	2.36	526	0.039	0.000
S_{14}	d_{yz}(153) → π^*_{tpy}(155) (MLCT)	87	2.46	504	0.028	0.000
S_{16}	d_{xy}(152) → π^*_{tpy}(155) (MLCT)<br>d_{yz}(153) → π^*_{tpy}(157) (MLCT)	78<br>16	2.51	494	0.021	0.000
S_{18}	d_{yz}(151) → π^*_{tpy}(157) (MLCT)<br>π^*_{tpy}(154) → π^*_{tpy}(166) (IL)<br>d_{yz}(151) → π^*_{tpy}(156) (MLCT)	45<br>22<br>12	2.57	483	0.034	0.000
S_{20}	d_{yz}(151) → π^*_{tpy}(155) (MLCT)<br>d_{xy}(152) → π^*_{tpy}(157) (MLCT)	60<br>24	2.61	475	0.115	0.000
S_{25}	π^*_{tpy}(154) → π^*_{tpy}(169) (IL)<br>π^*_{tpy}(154) → d_{z}(171) (LMCT)	51<br>34	3.13	396	0.199	0.000
S_{26}	π^*_{tpy}(154) → π^*_{tpy}(170) (IL)	88	3.18	390	0.144	0.000
**Doubly reduced triplet (S=1)**						
State	Transition	Weight / %	E^e / eV	λ / nm	f	$\langle s^2 \rangle$
T_9	π^*_{tpy}(154α) → π^*_{tpy}(160α) (IL)<br>π^*_{tpy}(155α) → π^*_{tpy}(161α) (IL)<br>π^*_{tpy}(155α) → π^*_{tpy}(159α) (IL)	51<br>31<br>15	1.58	785	0.010	2.048
T_{10}	π^*_{tpy}(155α) → π^*_{tpy}(160α) (IL)<br>π^*_{tpy}(154α) → π^*_{tpy}(159α) (IL)<br>π^*_{tpy}(154α) → π^*_{tpy}(161α) (IL)	46<br>27<br>24	1.59	779	0.016	2.044
T_{15}	π^*_{tpy}(155α) → π^*_{tpy}(163α) (IL)<br>π^*_{tpy}(154α) → π^*_{tpy}(164α) (IL)	43<br>36	2.12	584	0.048	2.138
T_{16}	d_{xy}(151β) → π^*_{tpy}(156β) (MLCT)<br>d_{yz}(153β) → π^*_{tpy}(157β) (MLCT)<br>d_{xz}(151α) → π^*_{tpy}(157α) (MLCT)	33<br>29<br>19	2.13	581	0.042	2.105
T_{32}	d_{xz}(151α) → π^*_{tpy}(157α) (MLCT)<br>d_{xy}(151β) → π^*_{tpy}(156β) (MLCT)<br>d_{xy}(151β) → π^*_{tpy}(154β) (MLCT)<br>d_{xz}(152β) → π^*_{tpy}(157β) (MLCT)<br>d_{xz}(152β) → π^*_{tpy}(155β) (MLCT)	35<br>15<br>13<br>11<br>9	2.56	484	0.037	2.105
T_{33}	d_{yz}(153β) → π^*_{tpy}(156β) (MLCT)<br>d_{xz}(152β) → π^*_{tpy}(156β) (MLCT)<br>d_{yz}(153β) → π^*_{tpy}(154β) (MLCT)<br>d_{xy}(152α) → π^*_{tpy}(157α) (MLCT)<br>d_{yz}(153α) → π^*_{tpy}(156α) (MLCT)	33<br>15<br>13<br>7<br>6	2.57	481	0.068	2.236
T_{35}	d_{xz}(152β) → π^*_{tpy}(156β) (MLCT)<br>d_{yz}(153β) → π^*_{tpy}(156β) (MLCT)<br>d_{xy}(151β) → π^*_{tpy}(157β) (MLCT)	56<br>21<br>7	2.61	476	0.083	2.211

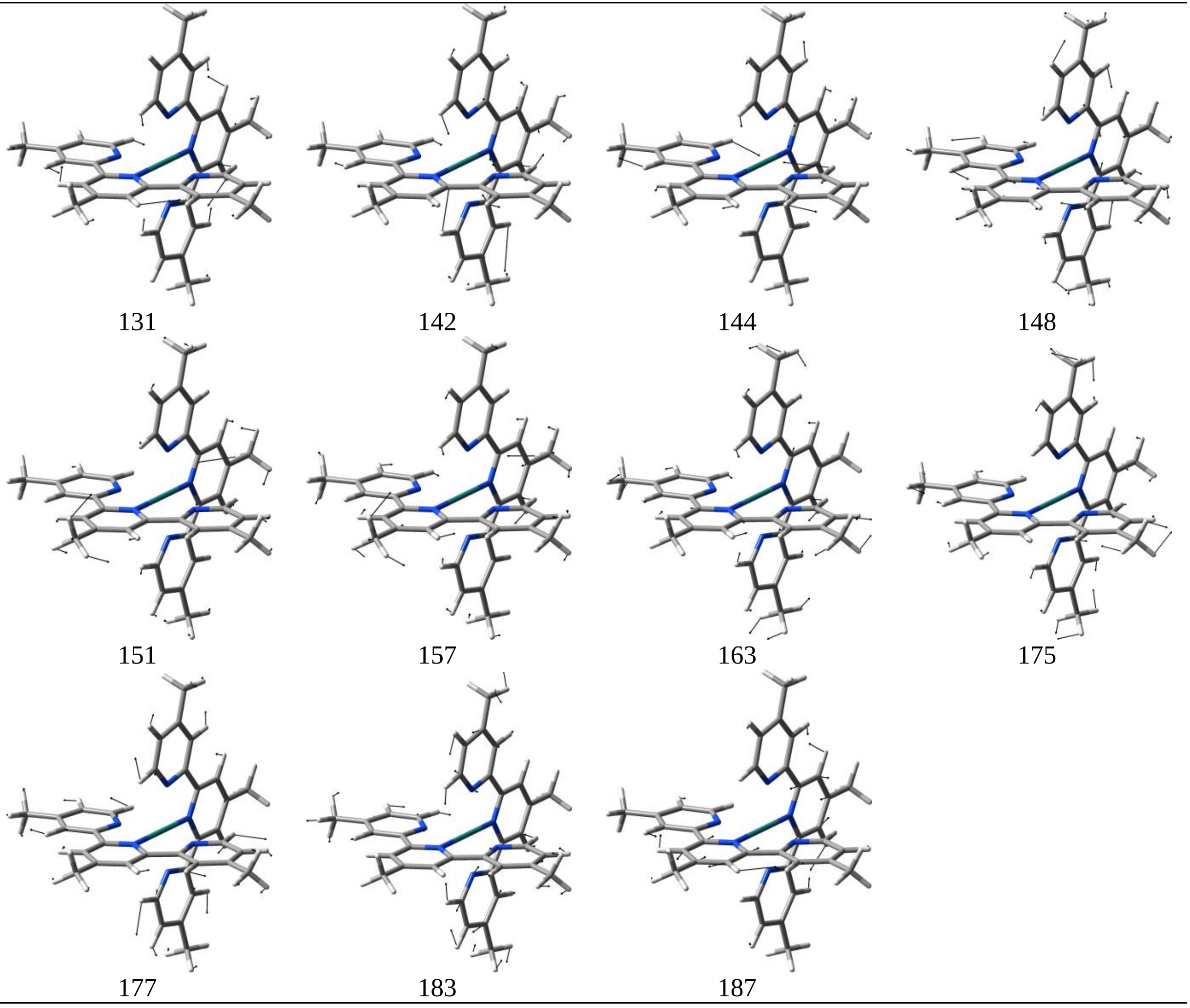

Figure S1: Resonance Raman active vibrational modes of non-reduced singlet species (S=0, λ_{exc} = 514 nm).

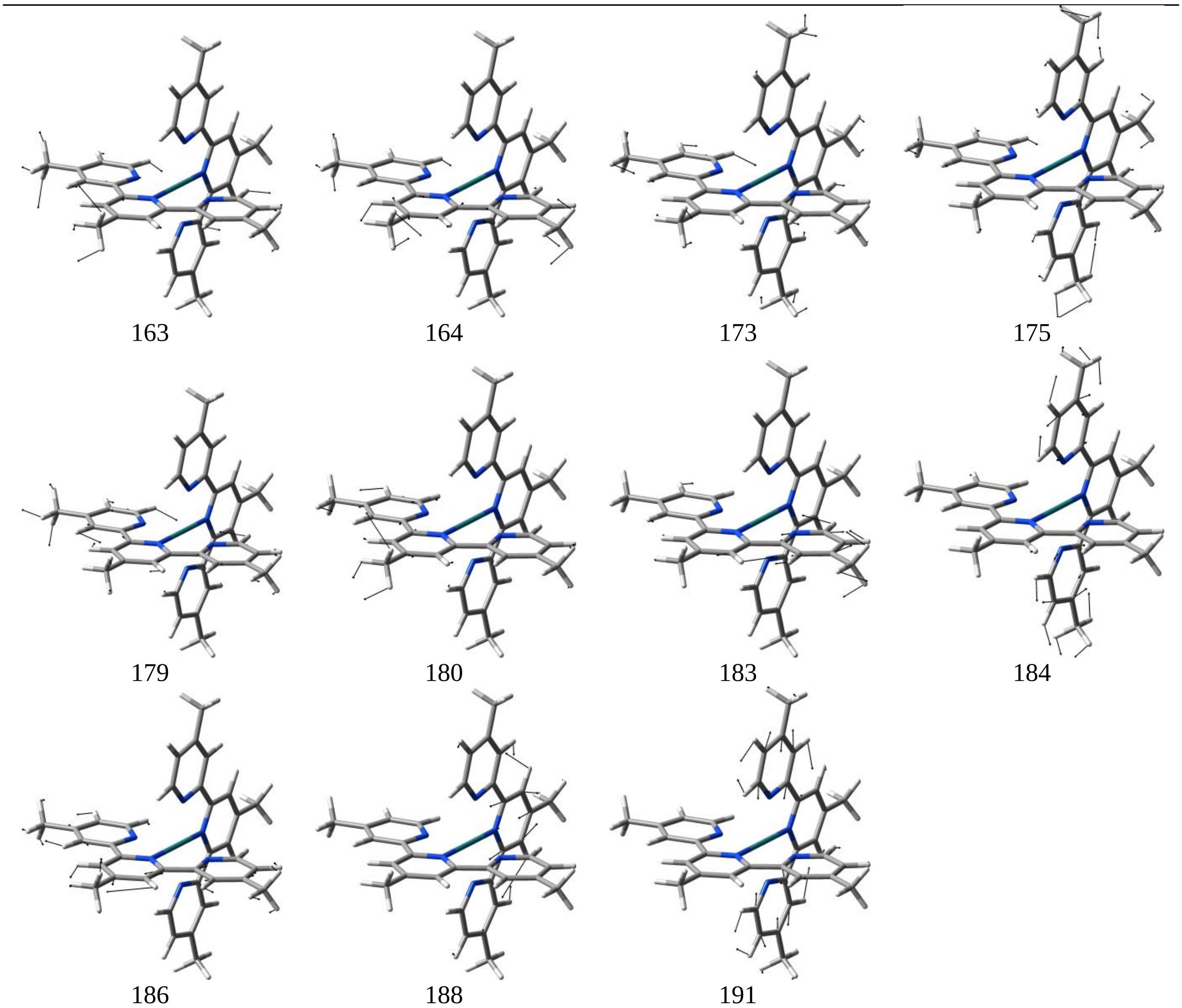

Figure S2: Resonance Raman active vibrational modes of singly reduced doublet species (S=1/2, λ_{exc} = 514 nm).

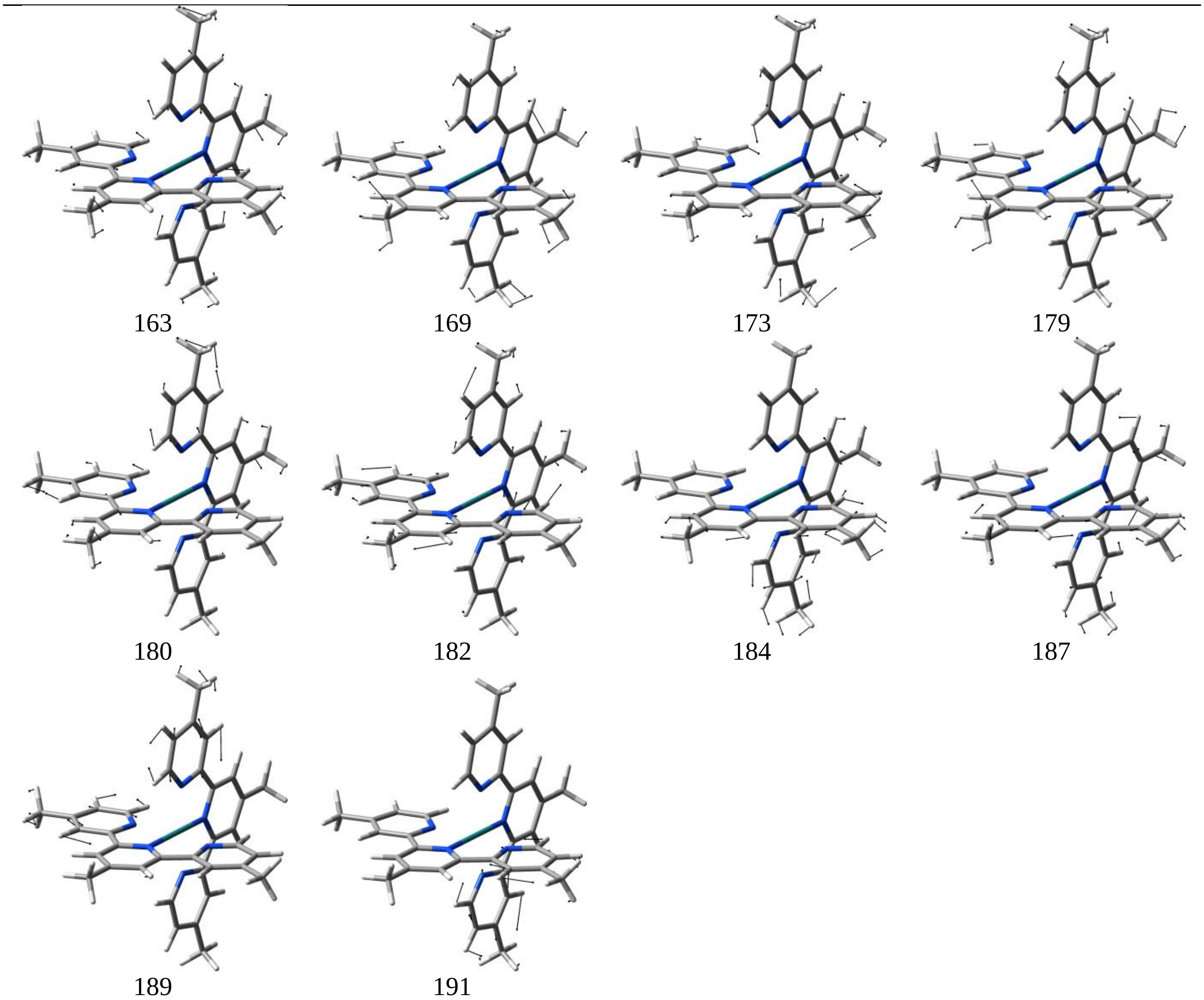

Figure S3 panels labelled 163, 169, 173, 179, 180, 182, 184, 187, 189, 191.

Figure S3: Resonance Raman active vibrational modes of doubly reduced triplet species (S=1, λ_{exc} = 514nm).

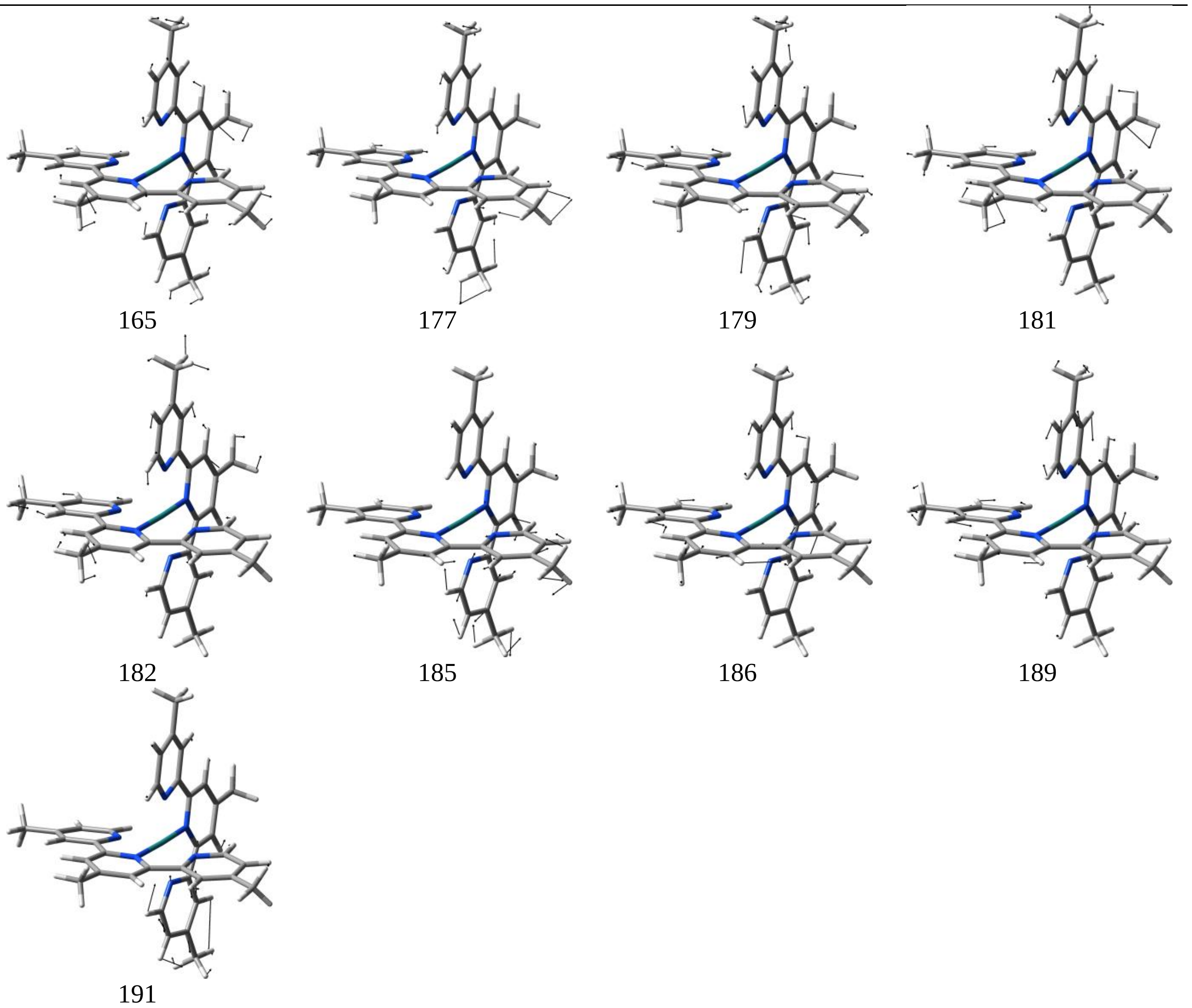

Figure S4: Resonance Raman active vibrational modes of doubly reduced singlet species (S=0, λ_{exc} = 514nm).

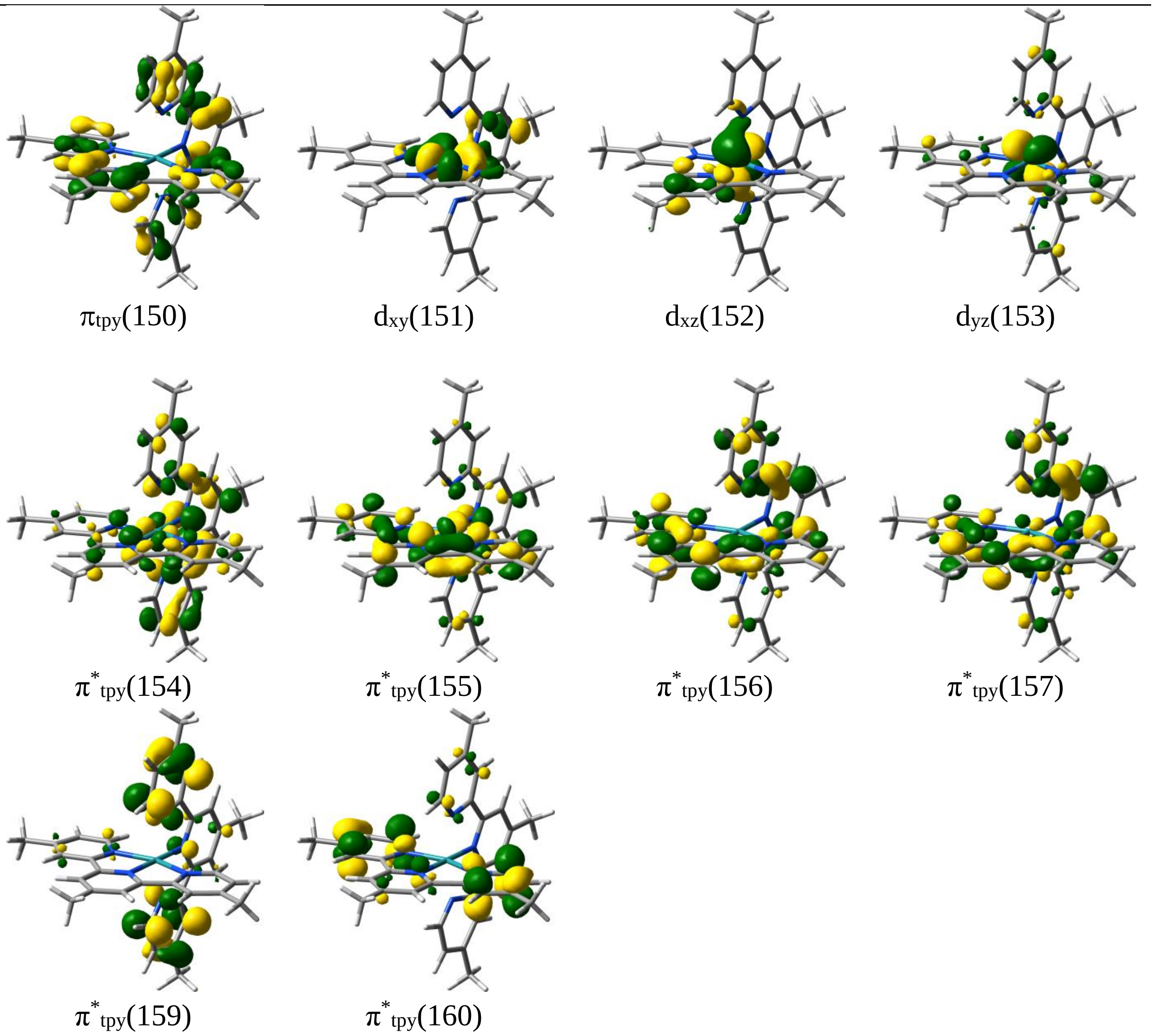

Figure S5: Molecular orbitals involved in the main transitions of the bright excited states calculated at the B3LYP/6-31G(d) level of theory for the non-reduced (S=0) ruthenium complex.

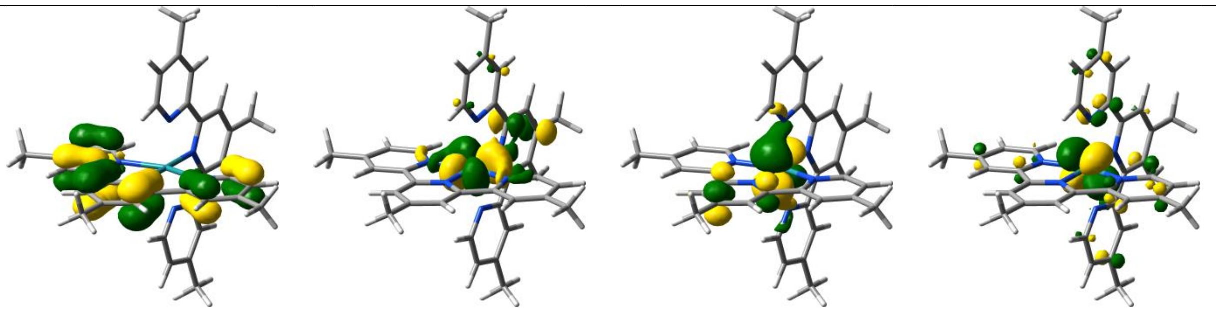

$\pi_{tpy}(149\alpha)$ $d_{xy}(151\alpha)$ $d_{xz}(152\alpha)$ $d_{yz}(153\alpha)$

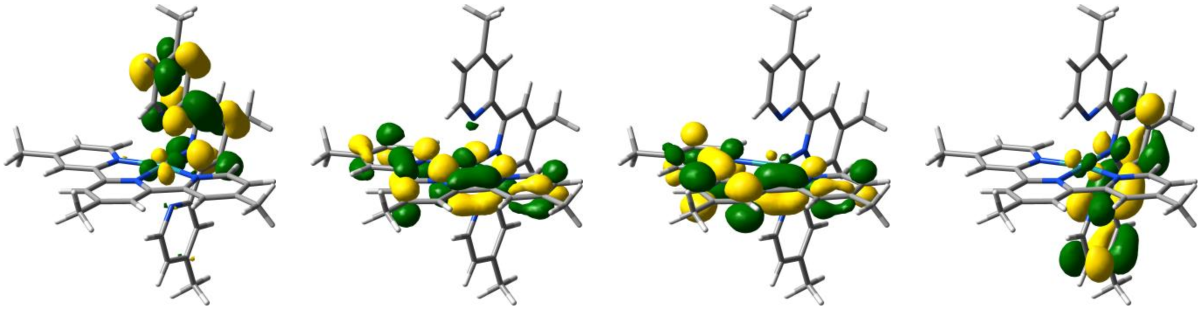

$\pi^*_{tpy}(154\alpha)$ $\pi^*_{tpy}(155\alpha)$ $\pi^*_{tpy}(156\alpha)$ $\pi^*_{tpy}(157\alpha)$

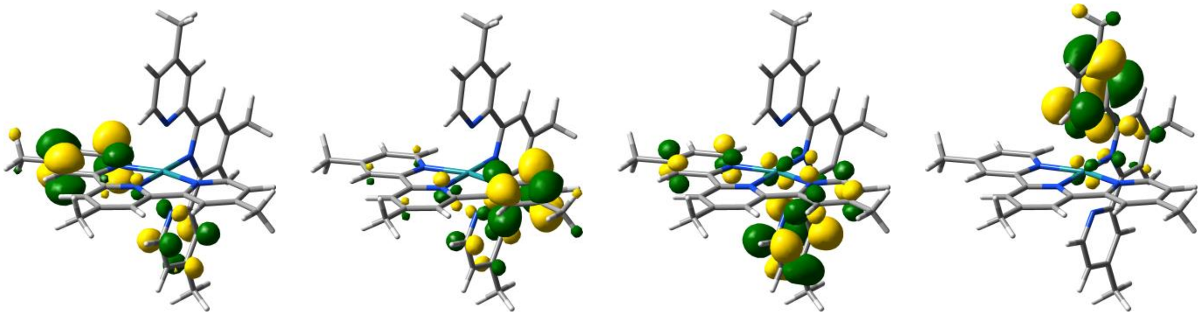

$\pi^*_{tpy}(158\alpha)$ $\pi^*_{tpy}(159\alpha)$ $\pi^*_{tpy}(160\alpha)$ $\pi^*_{tpy}(161\alpha)$

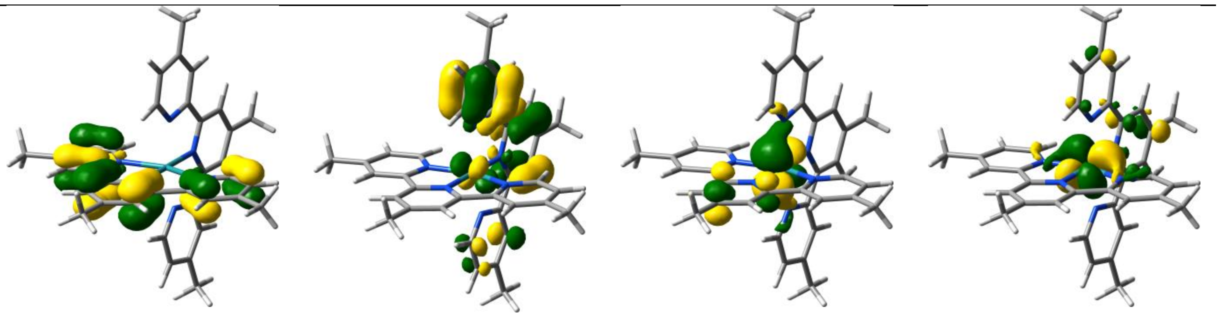

$\pi_{tpy}(149\beta)$ $\pi_{tpy}(150\beta)$ $d_{xz}(151\beta)$ $d_{xy}(152\beta)$

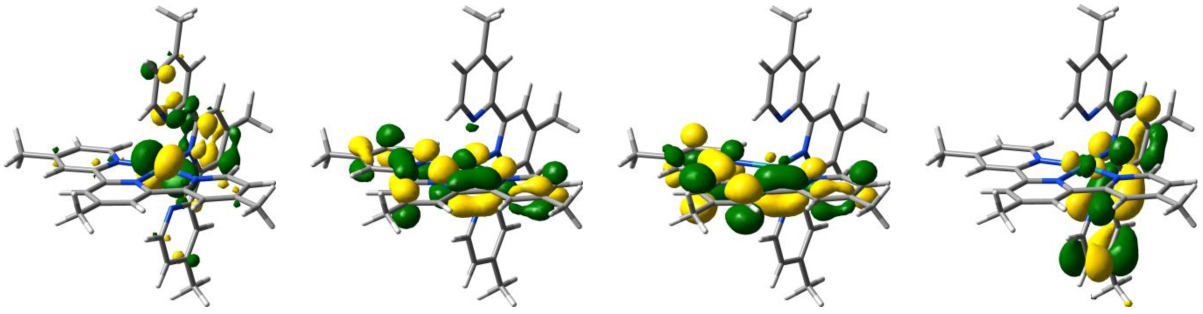

$d_{yz}(153\beta)$ $\pi^*_{tpy}(154\beta)$ $\pi^*_{tpy}(155\beta)$ $\pi^*_{tpy}(156\beta)$

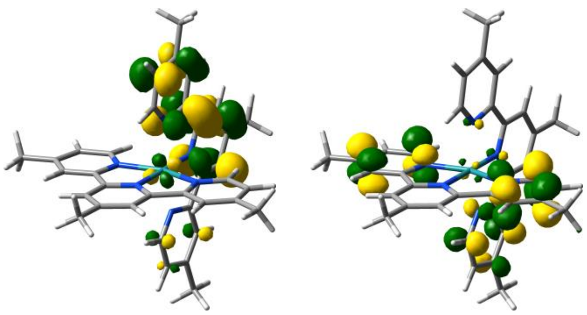

$\pi^*_{tpy}(157\beta)$ $\pi^*_{tpy}(159\beta)$

Figure S6: Molecular orbitals involved in the main transitions of the bright excited states calculated at the B3LYP/6-31G(d) level of theory for the singly reduced (S=1/2) ruthenium complex.

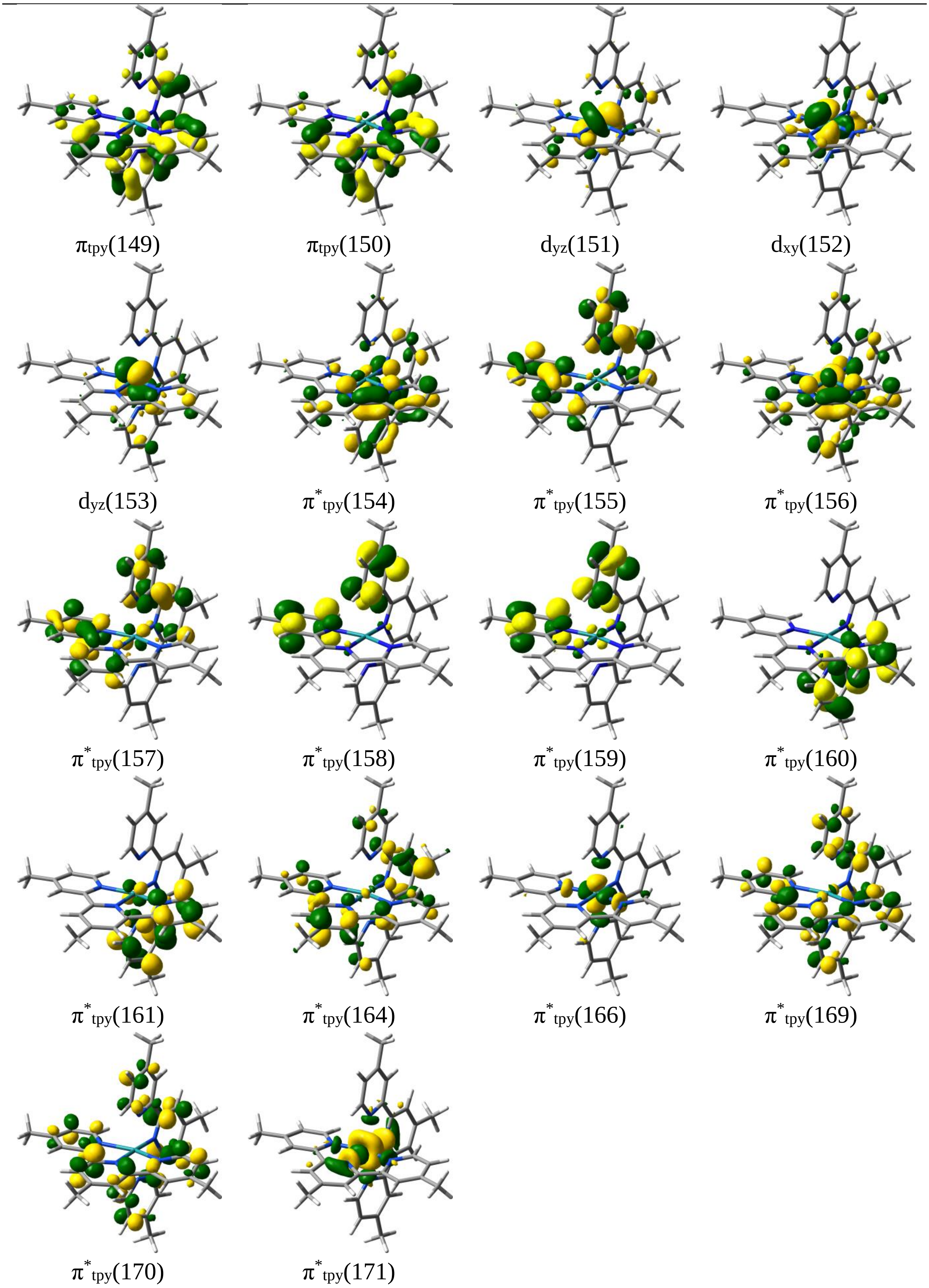

Figure S7: Molecular orbitals involved in the main transitions of the bright excited states calculated at the B3LYP/6-31G(d) level of theory for the doubly reduced (singlet multiplicity, S=0) ruthenium complex.

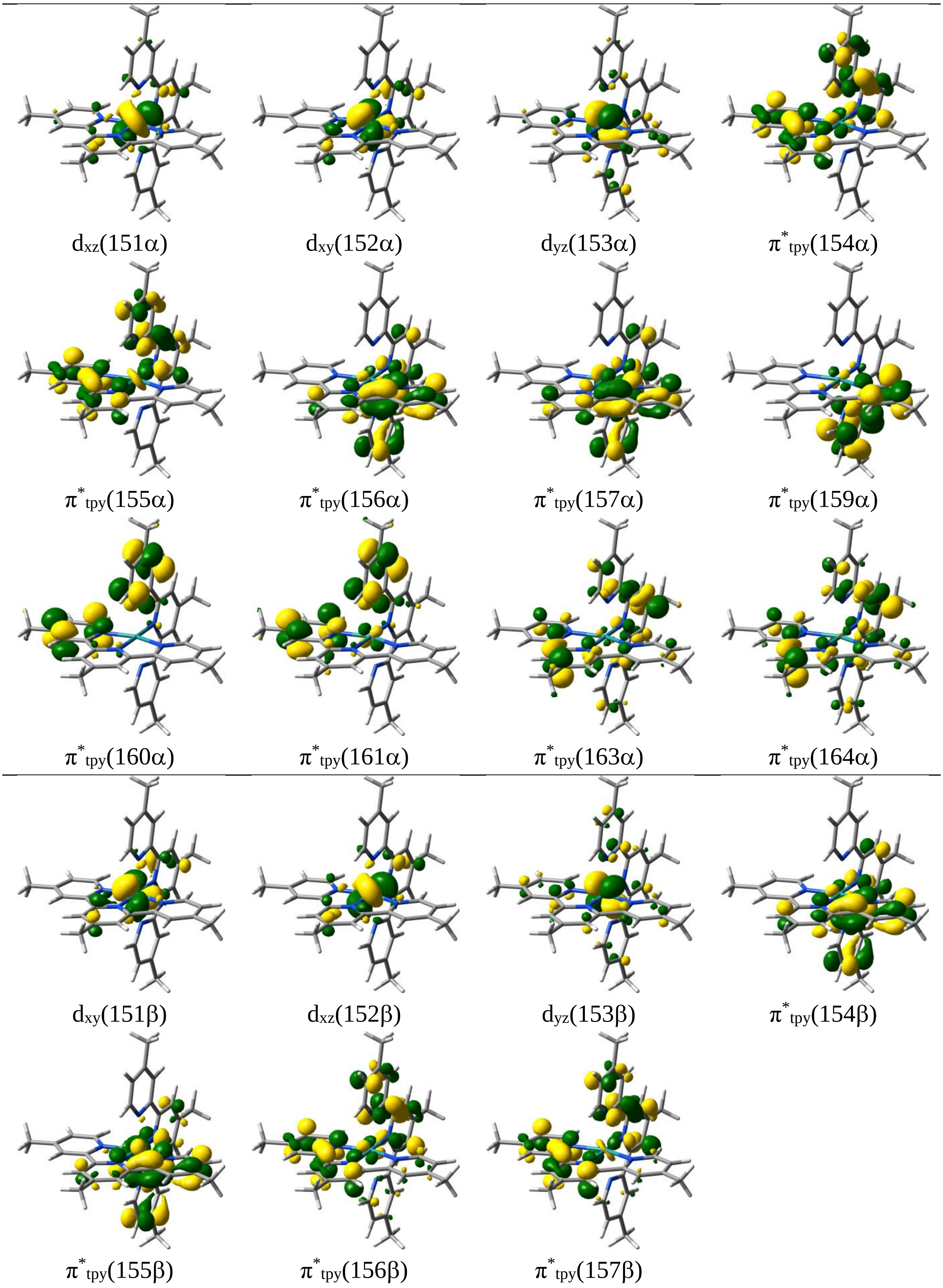

Figure S8: Molecular orbitals involved in the main transitions of the bright excited states calculated at the B3LYP/6-31G(d) level of theory for the doubly reduced (triplet multiplicity, S=1) ruthenium complex.

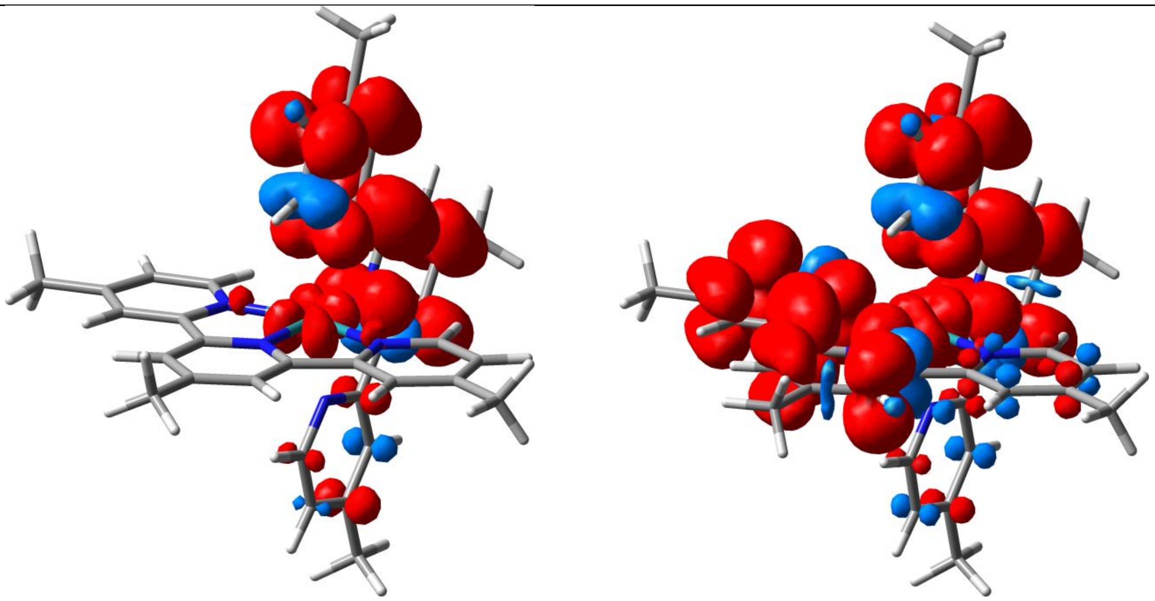

Singly reduced doublet | Doubly reduced triplet

Figure S9: Spin densities of the singly (doublet multiplicity, S=1/2) and doubly reduced (triplet multiplicity, S=1) ruthenium complex.
